# Physical Activity for Executive Function and Activities of Daily Living in AD Patients: A Systematic Review and Meta-Analysis

**DOI:** 10.3389/fpsyg.2020.560461

**Published:** 2020-12-03

**Authors:** Lin Zhu, Long Li, Lin Wang, Xiaohu Jin, Huajiang Zhang

**Affiliations:** ^1^School of Physical Education, Soochow University, Suzhou, China; ^2^Department of Physical Education, Wuhan University of Technology, Wuhan, China; ^3^College of Physical Education, Hubei University of Arts and Science, Xiangyang, China

**Keywords:** exercise prescription, ADL, executive function, AD, physical activity

## Abstract

**Objectives:** The present study aimed to systematically analyze the effects of physical activity on executive function, working memory, cognitive flexibility, and activities of daily living (ADLs) in Alzheimer's disease (AD) patients and to provide a scientific evidence-based exercise prescription.

**Methods:** Both Chinese and English databases (PubMed, Web of Science, the Cochrane Library, EMBASE, VIP Database for Chinese Technical Periodicals, China National Knowledge Infrastructure, and Wanfang) were used as sources of data to search for randomized controlled trials (RCTs) published between January 1980 and December 2019 relating to the effects of physical activity on executive function, working memory, cognitive flexibility, and ADL issues in AD patients. Sixteen eligible RCTs were ultimately included in the meta-analysis.

**Results:** Physical activity had significant benefits on executive function [standard mean difference (SMD) = 0.42, 95% confidence interval (CI) 0.22–0.62, *p* < 0.05], working memory (SMD = 0.28, 95% CI 0.11–0.45, *p* < 0.05), cognitive flexibility (SMD = 0.23, 95% CI −0.02 to 0.47, *p* < 0.01), and ADLs (SMD = 0.68, 95% CI 0.19–1.16, *p* < 0.05) among AD patients. Subgroup analysis indicated that, for executive function issues, more than 60 min per session for 16 weeks of moderate-to-high-intensity dual-task exercises or multimodal exercise had a greater effect on AD patients. For working memory and cognitive flexibility issues, 60–90 min of moderate-intensity dual-task exercises 1–4 times/week was more effective. For ADL issues, 30–90 min of multimodal exercise at 60–79% of maximal heart rate (MHR) 3–4 times/week had a greater effect on AD patients.

**Conclusions:** Physical activity was found to lead to significant improvements in executive function, working memory, cognitive flexibility, and ADLs in AD patients and can be used as an effective method for clinical exercise intervention in these patients. However, more objective, scientific, and effective RCTs are needed to confirm this conclusion.

## Introduction

Alzheimer's disease (AD) is a common disorder of the nervous system, accounting for disease in 60–70% of patients with dementia (Reitz et al., 2011), causing severe clinical, social, and economic problems (Prince et al., 2013). Features of dementia include progressive cognitive decline, including loss of memory, language, or executive function, and subsequent decline of social function, for example, activities of daily living (ADLs) (Allal et al., 2015). The cognitive process of the frontal lobe may also change, which is characterized by decreased attention and executive function, as evidenced by deficits in problem solving, planning, and organizing behavior and ideas, abstraction, judgment, cognitive flexibility, decision making, working memory, and self-monitoring (Avilla and Miotto, 2002; Yaari and Bloom, 2007). By 2050, the number of people ≥60 years old will increase by 1.25 billion (Prince et al., 2013), and there will be an estimated 115.4 million people with dementia (Maffei et al., 2017). Drug therapy has been shown to be beneficial to the cognitive function and dependence in ADLs in AD patients (Tan et al., 2014), but it also has side effects.

Based on the above factors, alternative treatment options for AD are necessary to achieve better treatment results. Some research shows that approximately one-third of all AD cases may be due to potentially modifiable factors, such as lack of physical activity (Norton et al., 2014), which means that the disease can be prevented. Furthermore, some human and animal studies have shown that physical activity can promote the improvement of cerebrovascular function, perfusion, and brain neural plasticity, which can prevent the gradual loss of cognitive function or executive function related to diseases, such as aging and dementia (Davenport et al., 2012; Erickson et al., 2012). Furthermore, physical activity is considered to have a significant effect on executive function (Wilbur et al., 2012), as confirmed recently in a large experiment of moderate-intensity exercise in sedentary older adults: in the subgroup with the weakest cognitive ability, executive function was improved. In recent years, more and more studies have confirmed the positive effect of physical activity among AD patients. Meanwhile, executive function can directly affect the ADLs or continued independence (Royall et al., 2000; Bell-McGinty et al., 2002; Cahn-Weiner et al., 2002). Some studies provide support for the hypothesis that commonly used clinical trials of executive function significantly predict the ADLs (Bell-McGinty et al., 2002; Cahn-Weiner et al., 2002). As such, with a decrease in executive function, a breakdown in successful execution and completion of complex behavioral procedures is likely, especially in subsets of ADLs involving executive control (Bell-McGinty et al., 2002). However, some ADLs (e.g., ambulating, cooking, reading, leisure, housework, and managing finances) promote improved physical, cognitive, and executive functions (Bell-McGinty et al., 2002; Cahn-Weiner et al., 2002; Jekel et al., 2015). More and more studies have shown that physical activity has a significant impact on improving executive function and ADL issues in AD patients, thus improving their quality of life. In addition, the World Health Organization (WHO) recommends that people over the age of 65 should take at least 150 min of moderate-intensity aerobic exercise (such as brisk walking and jogging) every week, 75 min of high-intensity aerobic exercise every week, or a combination of the two supplemented by muscle-strengthening activities (such as resistance exercise and stretching exercise) on 2 or more days every week (World Health Organization Physical Activity Older Adults, 2017).

Recently, a great deal of research has been carried out to evaluate the impact of physical activity on executive function or ADL issues among AD patients. Because of the differences in the intervention samples, timing, frequency, intensity, and duration, the specific effects on executive function and ADL issues among AD patients could have been different. Therefore, the aim of our meta-analysis was to evaluate the impact of physical activity on executive function and ADL issues in AD patients. Moreover, executive function is a complex construct that includes different functions, such as cognitive flexibility inhibition and working memory. However, there are few studies on inhibitory functions in AD patients. Therefore, this study assessed the specific effects of physical activity on cognitive flexibility and working memory issues in AD patients. This study also explored the internal regulation mechanism of physical activity on the executive functions of AD patients to provide a corresponding exercise prescription.

## Methods

### Search Strategy

Literature was identified using the following databases: PubMed, Web of Science, the Cochrane Library, EMBASE, VIP Database for Chinese Technical Periodicals, China National Knowledge Infrastructure, and Wanfang. These databases were searched to identify randomized controlled trials (RCTs) published in any language between January 1, 1980 and December 31, 2019. The search terms used included “exercise or physical activity or aerobic exercise or physical exercise or aerobic fitness or walking or cycling or strength training or balance training or flexibility training” with AD terms including “AD or Alzheimer's disease or Alzheimer” as well as “executive function or executive functions or ADL or activities of daily living.”

### Inclusion Criteria

The selection criteria were as follows: (1) RCTs investigating the impact of any type of physical activity as an additional intervention on executive function or ADLs; (2) sample population including a group of old people (aged ≥50 years) and participants diagnosed with Alzheimer's-type dementia of any severity, excluding diagnoses of other dementias or mild cognitive impairment (MCI); (3) interventions in an experimental group involving physical activity (e.g., aerobic exercise, aerobic fitness, walking, cycling, strength training, balance training, and flexibility training) compared with different types of control groups (e.g., usual care, no physical activity, and no-intervention control group); (4) outcome indicators including test data on executive function and ADLs; and (5) publication language of Chinese or English.

### Exclusion Criteria

The exclusion criteria were as follows: (1) duplicated studies; (2) reviews, observational studies, abstract-only articles (without full-text article available), and non-RCT studies; and (3) studies with no data or unclear data reported for analysis.

### Collection of Studies

Two investigators (LZ and LW) independently reviewed the titles and abstracts from the search results and screened out the full texts that might meet the criteria. If a study met the inclusion criteria, it received a full-text article evaluation. When there was any disagreement between the two reviewers, a third reviewer (LL) was invited to discuss with them and to verify the eligibility of the uncertain article. All eligible studies included information, such as author, publication year, country, sample size, sample population age, intervention methods, duration, measurement standards, experimental results, and dropouts.

### Data Extraction

Detailed information included the first author, publication year, participant characteristics (sample size and age range/mean age), intervention design (frequency, duration of each intervention session, duration, and follow-up), outcome measure, statistical analyses, and results. Meanwhile, we also extracted quantitative data from the research results: mean and standard deviation (SD) of executive function and ADLs between physical activity and usual care, including its corresponding sample size.

### Methodological Quality Assessment

Two authors used the modified the Physical Therapy Evidence Database (PEDro) scale (Zou et al., 2019) to independently perform methodological quality assessment of each eligible study. This assessment consisted of nine items (randomization, concealed allocation, similar baseline, blinding of assessors, ≤15% dropouts, intention-to-treat analysis, between-group comparison, point measure and measures of variability, and isolate exercise intervention), and higher scores indicate better quality of the method.

### Statistical Analysis

Stata 14.0 (StataCorp, Texas, USA) was used to calculate effect sizes [standardized mean difference (SMD)] of physical activity on executive function and ADLs. SMD was considered as small (0.2–0.49), moderate (0.5–0.79), or large (0.8). According to the intervention system review of the Cochrane Collaboration handbook, selection of fixed-effects or random-effects meta-analysis should be based on the actual effect of an intervention on outcome measures. Differences (standard mean difference, SMD) and 95% confidence intervals (95% CIs) were calculated. *I*^2^ values of 25, 50, and 75% are considered to be low, medium, and high heterogeneity (Higgins et al., 2003). When the heterogeneity test *I*^2^ ≥ 50%, a random-effects model was used for meta-analysis. In this study, regression analysis was used to study the degree of experimental heterogeneity. Subgroup analyses were performed according to categorical variables, including sample age, exercise intensity, frequency, duration, duration of each intervention session, and exercise type. Subgroup analysis was used to determine which subgroup was more effective for improving the executive function and ADLs among AD patients.

## Results

### Study Selection

**Figure 1:**
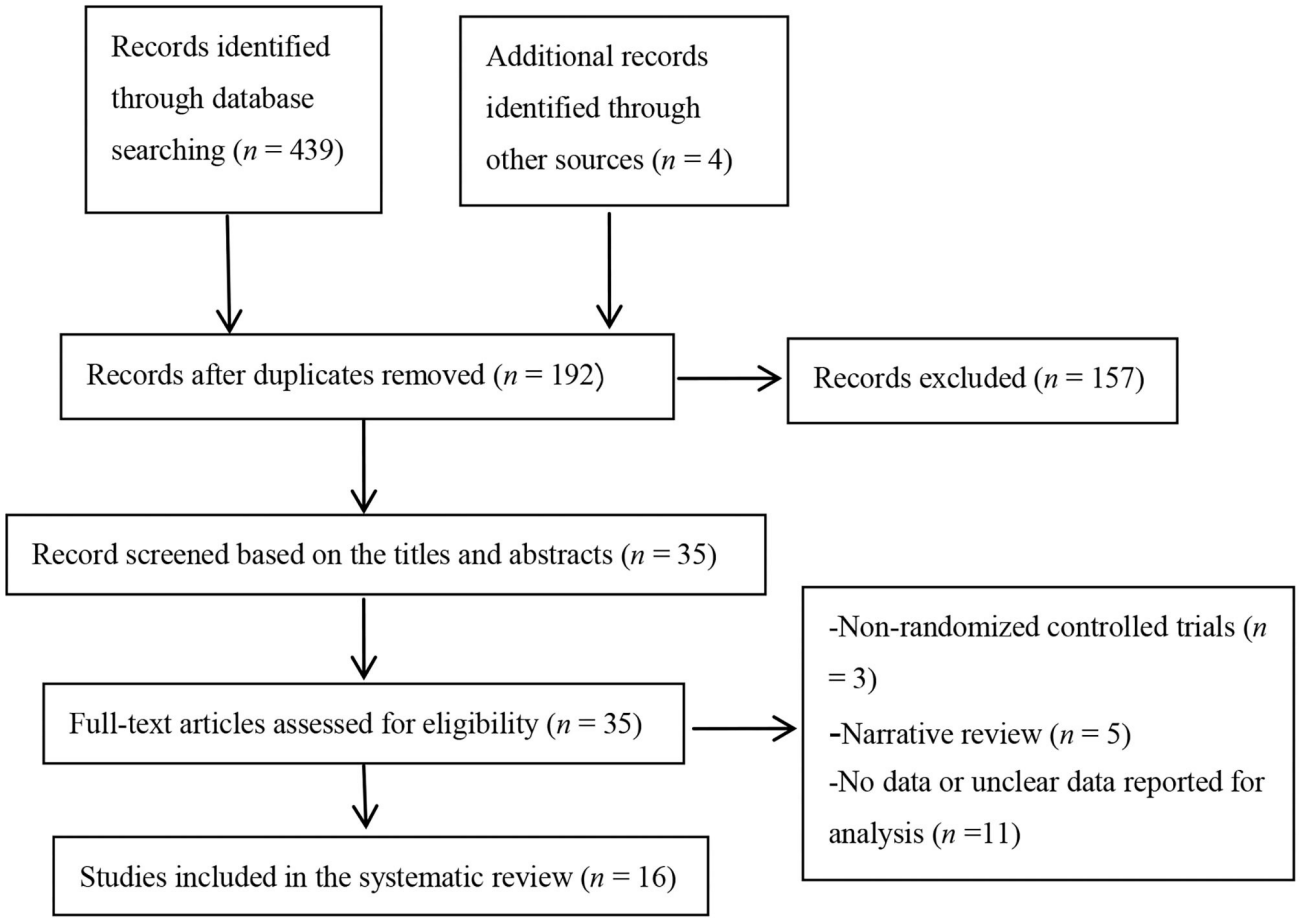
Flowchart of study selection.

A total of 443 topic-related articles were identified from 7 databases and other resources (Figure 1). After removing duplicate articles, 192 articles remained. A total of 157 articles were deleted after titles and abstracts were screened for non-related articles (*n* = 148) and abstract-only articles (*n* = 9). The remaining 35 articles were further screened after reading the full-text articles. Nineteen studies were removed because they were non-RCTs (*n* = 3), reviews (*n* = 5), or had no or unclear outcome measures (*n* = 11). Finally, our meta-analysis included 16 eligible studies.

### Characteristics of Eligible Studies

There were 16 eligible (Venturelli et al., 2011; Coelho et al., 2012; Vreugdenhill et al., 2012; Andrade et al., 2013; Chang et al., 2015; Holthoff et al., 2015; El-Kader and Al-Jiffr, 2016; Hoffmanna et al., 2016; Ohman et al., 2016; Morris et al., 2017; Pedroso et al., 2017; Rolland et al., 2017; Tootsa et al., 2017; Vidoni et al., 2017; Fonte et al., 2019; Silvaa et al., 2019) RCTs, as shown in Table 1. There were 1,181 participants in total; the smallest sample was 27 participants (Silvaa et al., 2019), and the largest sample was 200 participants (Holthoff et al., 2015). The age of the participants in the experiment ranged from 50 to 96 years old. The shortest experimental period was 8 weeks (El-Kader and Al-Jiffr, 2016), and the longest experimental period was 1 year (Rolland et al., 2017). The experimental group included various interventions, such as moderate-to-high-intensity physical activity, ergometer bicycle exercise, walking, cycling, strength training, balance training, and flexibility training. The control group was treated by usual care, standard treatment, routine medical care, etc. (Table 1).

**Table 1:** Summary characteristics of the included studies.

**References**	**Country**	**Sample size (attrition rate)**	**Mean age or age range**	**Duration (W)**	**Experimental group intervention**	**Control group intervention**	**Outcome assessments**	**Follow-up**
Morris et al. (2017)	US	76 (10.5%)	T = 74.4 (6.7) C = 71.4 (8.4)	26	AEx: 150 min/week of moderate-intensity aerobic exercise (cycling, walking, arm cranking on a specific ergometer)	ST: stretching and toning control program	EF	No
Fonte et al. (2019)	Italy	41 (0%)	T = 79 (9) C = 79 (7)	24	3 × 90 min/week moderate-intensity endurance and resistance training	Standard treatment	FAB/IADL	Yes
Ohman et al. (2016)	Helsinki	140 (21%)	T = 77.7 (5.4) C = 78.1 (5.3)	48	2 × 60 min/week executive function-related exercises, dual-task training, and balance, endurance, or aerobic exercises	Usual care	CDT/VF	No
Tootsa et al. (2017)	Sweden	186 (13.4%)	T = 84.4 (6.2) C = 85.9 (7.8)	16	3 × 45 min/week high-intensity functional exercise (HIFE) program (weight-bearing exercises, strength exercises, balance training)	Regular daily life	VF	No
Pedroso et al. (2017)	Brazil	42 (14.2%)	T = 77.6 (6.2) C = 79.2 (5.6)	12	3 × 60 min/week functional-task training: warm-up exercise period (walking, stretching exercises) + stimulated locomotion (walking up and down the stairs, zigzag jogging, etc.) + stimulate other activities of daily living (sitting down and getting up, moving objects)	Standard medical care	VF/DAFS-R	No
Holthoff et al. (2015)	Germany	30 (0%)	T = 72.4 (4.3) C = 70.6 (5.4)	12	3 × 30 min/week PA intervention program: motor-assisted or active resistance training of the legs on a movement trainer	Usual care	EF/ADCS-ADLs	Yes
Coelho et al. (2012)	Brazil	27 (0%)	T = 78 (7.3) C = 77.1 (7.4)	16	3 × 60 min/week multimodal exercise (strength/resistance exercises, agility, flexibility, strength, balance, and cognitive training)	Regular daily life	FAB/CDT	No
Silvaa et al. (2019)	Brazil	27 (0%)	T = 81.2 (8.8) C = 77.5 (8.0)	12	2 × 60 min/week multimodal training session (balance training, aerobic exercise, strength training)	Usual care	CDT/VF	No
Andrade et al. (2013)	Brazil	30 (0%)	T = 78.6 (7.1) C = 77.0 (6.3)	16	3 × 60 min/week multimodal exercise (warm-up, aerobic work, dual-task activities)	Usual care	EF/CDT	No
El-Kader and Al-Jiffr (2016)	Saudi Arabia	59 (32.2%)	T = 68.9 (5.7) C = 69.1 (6.1)	8	3 × 45 min/week aerobic exercise (warm-up, stretching exercises, aerobic exercise, cooling down [on treadmill with low speed and without inclination])	Usual treatment	SF-36PF	No
Rolland et al. (2017)	France	134 (17.9%)	T = 82.8 (7.8) C = 83.1 (7.0)	48	2 × 60 min/week aerobic, strength, flexibility, and balance training	Routine medical care	ADLs	No
Vreugdenhill et al. (2012)	Australia	40 (0%)	T = 73.5 (51–83) C = 74.7 (58–89)	16	7 × 30 min/week strength and balance training and brisk walking + usual treatment	Usual treatment	Barthel Index/IADL	Yes
Venturelli et al. (2011)	Italy	24 (14.3%)	T = 83 (6) C = 85 (5)	24	4 × 30 min/week moderate exercise (walking)	Routine care	Barthel Index	No
Vidoni et al. (2017)	Italy	65 (0%)	T = 74.1 (6.8) C = 71.1 (8.8)	26	AEx: 150 min/week of moderate-intensity aerobic exercise	ST: stretching and toning control program	BADL/IADL	No
Hoffmanna et al. (2016)	Denmark	200 (5%)	T = 69.8 (7.4) C = 71.3 (7.3)	16	3 × 30 min/week moderate-to-high-intensity aerobic exercise (ergometer bicycle, cross trainer, treadmill)	Usual treatment	SDMT/ADCS-ADLs	No
Chang et al. (2015)	China	60 (5%)	T = 70.7 (7.4) C = 70.2 (8.5)	16	3 × 60–90 min/week cycling or treadmill + routine medical care	Routine medical care	ADCS-ADLs	No

*W, week; EF, executive function; AEx, aerobic exercise condition; ST, stretching and toning control condition; FAB, Frontal Assessment Battery; IADL, instrumental activity of daily living; CDT, clock-drawing test; VF, verbal fluency test; DAFS-R, Direct Assessment of Functional Status; ADCS-ADLs, Alzheimer's Disease Cooperative Study—Activities of Daily Living; SDMT, Symbol Digit Modalities Test; SF-36PF, SF-36: Physical Functioning; BADL, basic instrumental activities of daily living*.

### Methodological Quality Assessment

The methodological quality score for all qualified studies was between 5 and 8 (Table 2). All studies were RCTs and had similar baseline characteristics, between-group comparisons, point measures, measures of variability description, and isolated exercise interventions. Only four studies had concealed allocations and blinding of assessors (Venturelli et al., 2011; Rolland et al., 2017; Tootsa et al., 2017; Fonte et al., 2019). The dropout rates in three of these studies were all higher than 15% (El-Kader and Al-Jiffr, 2016; Ohman et al., 2016; Rolland et al., 2017), and only one study used the intention-to-treat principle (Rolland et al., 2017).

**Table 2:** Quality evaluation of eligible randomized controlled trials.

**References**	**Item 1**	**Item 2**	**Item 3**	**Item 4**	**Item 5**	**Item 6**	**Item 7**	**Item 8**	**Item 9**	**Score**
Morris et al. (2017)	1	0	1	0	1	0	1	1	1	6
Fonte et al. (2019)	1	1	1	1	1	0	1	1	1	8
Ohman et al. (2016)	1	0	1	0	0	0	1	1	1	5
Tootsa et al. (2017)	1	1	1	1	1	0	1	1	1	8
Pedroso et al. (2017)	1	0	1	0	1	0	1	1	1	6
Holthoff et al. (2015)	1	0	1	0	1	0	1	1	1	6
Coelho et al. (2012)	1	0	1	0	1	0	1	1	1	6
Silvaa et al. (2019)	1	0	1	0	1	0	1	1	1	6
Andrade et al. (2013)	1	0	1	0	1	0	1	1	1	6
El-Kader and Al-Jiffr (2016)	1	0	1	0	0	0	1	1	1	5
Rolland et al. (2017)	1	1	1	1	0	1	1	1	1	8
Vreugdenhill et al. (2012)	1	0	1	0	1	0	1	1	1	6
Venturelli et al. (2011)	1	1	1	1	1	0	1	1	1	8
Vidoni et al. (2017)	1	0	1	0	1	0	1	1	1	6
Hoffmanna et al. (2016)	1	0	1	0	1	0	1	1	1	6
Chang et al. (2015)	1	0	1	0	1	0	1	1	1	6

*Item 1, randomization; Item 2, concealed allocation; Item 3, similar baseline; Item 4, blinding of assessors; Item 5, <15% dropouts; Item 6, intention-to-treat analysis; Item 7, between-group comparison; Item 8, point measure and measures of variability; Item 9, isolate exercise intervention; 1, explicitly described and present in details; 0, absent, inadequately described, or unclear*.

### Meta-Analysis of Outcome Indicators

#### Effect of Physical Activity on Executive Function Issues in AD Patients

**Figure 2:**
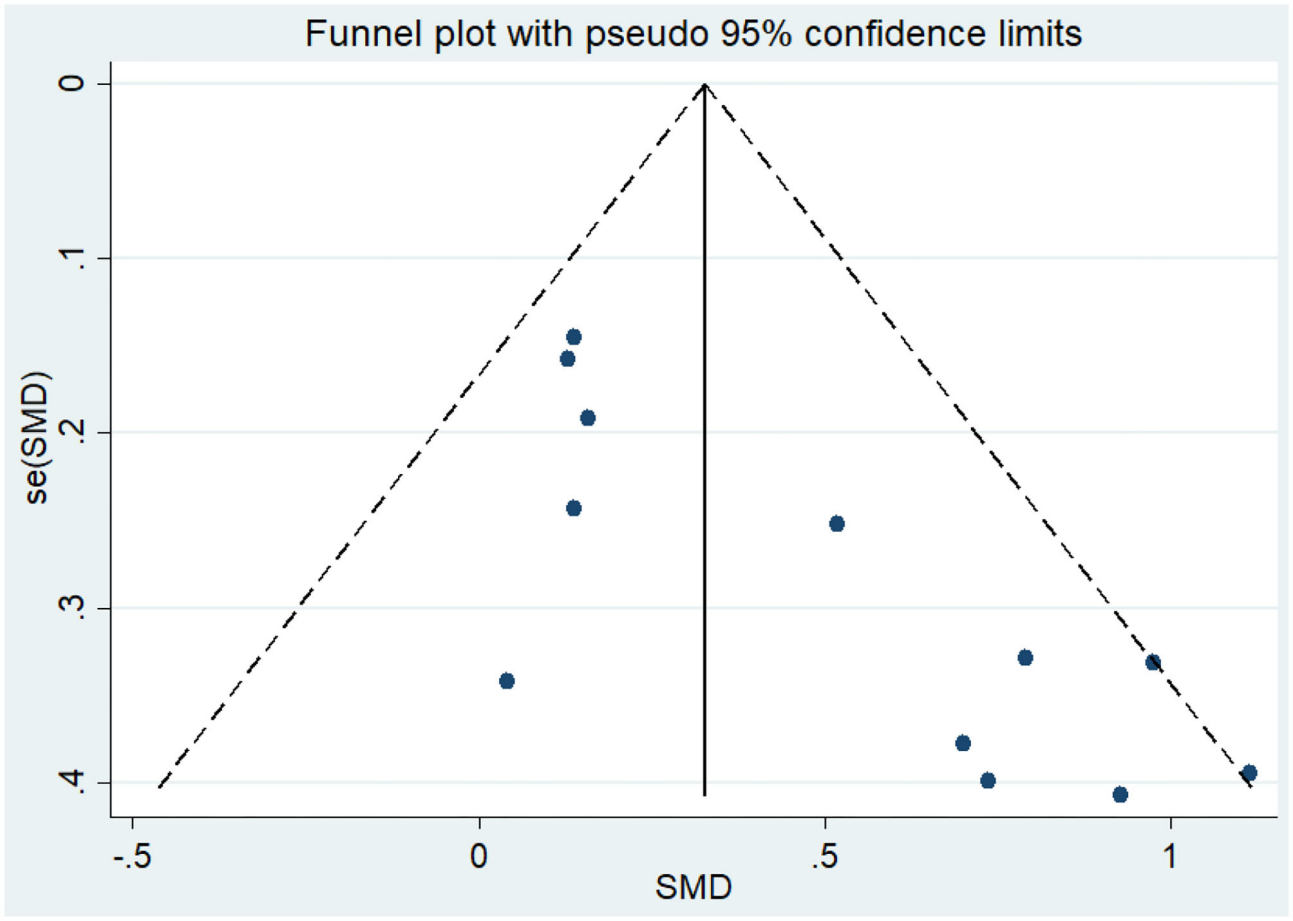
Funnel plot of publication bias for executive function.

**Figure 3:**
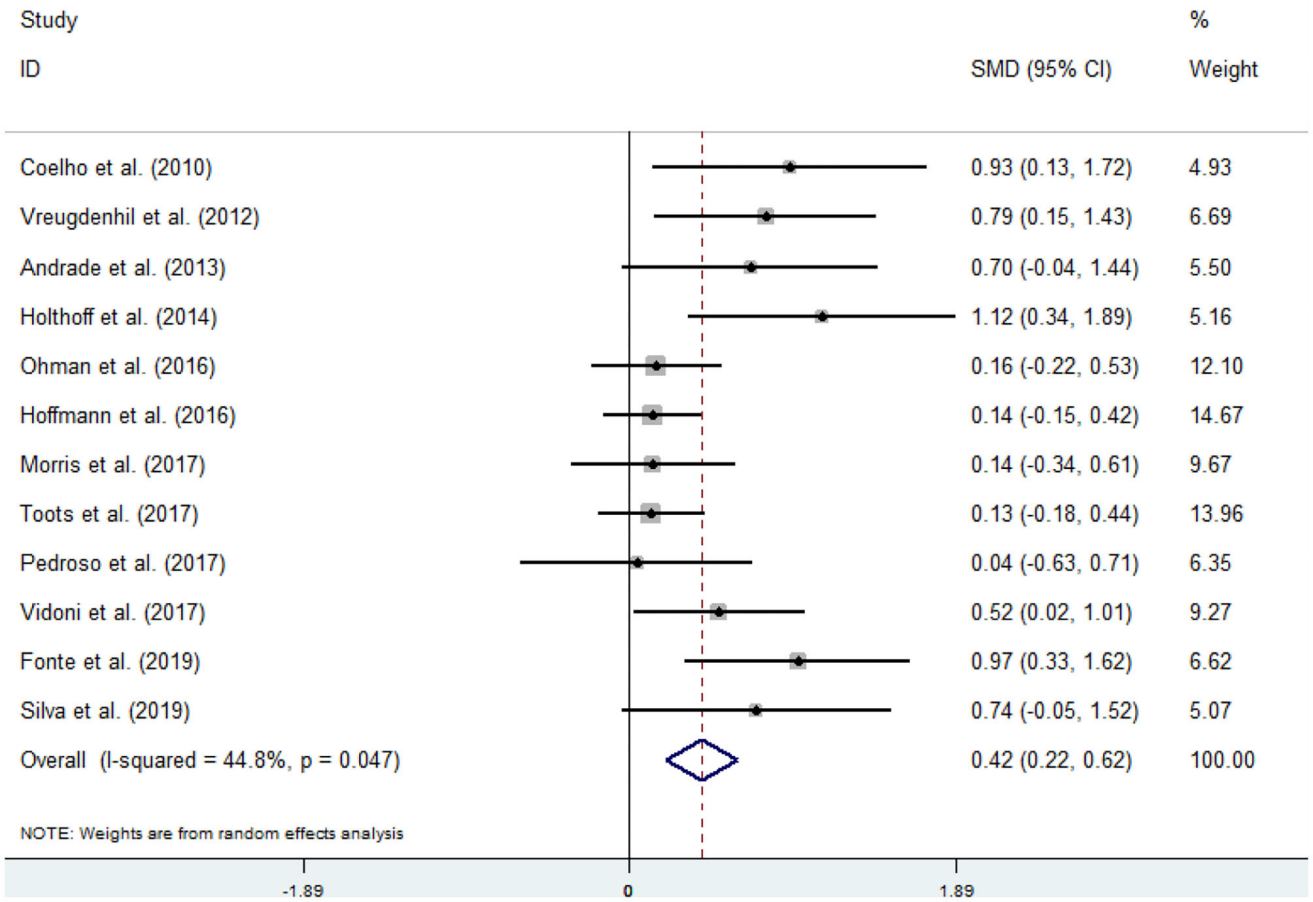
Effect of physical activity on executive function.

Twelve articles (Coelho et al., 2012; Vreugdenhill et al., 2012; Andrade et al., 2013; Holthoff et al., 2015; Hoffmanna et al., 2016; Ohman et al., 2016; Morris et al., 2017; Pedroso et al., 2017; Tootsa et al., 2017; Vidoni et al., 2017; Fonte et al., 2019; Silvaa et al., 2019) compared the effects of an intervention group and a control group on executive function before and after the experiment. An asymmetrical funnel plot was presented. The funnel plot shows that there were no outlier values (Figure 2). There was a moderate heterogeneity in the research literature (*p* = 0.047, *I*^2^ = 44.8%), and a random-effects model was selected for the meta-analysis (Figure 3). The meta-analysis of 12 studies demonstrated that physical activity had significant effects on improving executive function in AD patients (SMD = 0.42, 95% CI 0.22–0.62, *p* < 0.05).

Covariates including age, intensity, frequency, time, and duration are likely to be the influencing factors for executive function issues in AD patients. The results of the regression of covariates for executive function issues in AD patients are presented in Table 3. For executive function, there were no significant effects of age (95% CI −0.0423797 to 0.0645279, *p* = 0.63), intensity (95% CI −0.0572985 to 0.0029897, *p* = 0.07), frequency (95% CI −0.131867 to 0.3570966, *p* = 0.303), time (95% CI −0.0040595 to 0.0114874, *p* = 0.287), or duration (95% CI −0.0562039 to 0.0070974, *p* = 0.106).

**Table 3:** Covariate regression analysis of executive function issues in AD patients.

**_ES**	**Coef**.	**Std. err**.	***t***	***p* > *t***	**(95% CI)**	
Age	0.0110741	0.0218454	0.51	0.63	−0.0423797	0.0645279
Intensity (%)	−0.0271544	0.0123192	−2.20	0.07	−0.0572985	0.0029897
Frequency (times/week)	0.1126148	0.0999144	1.13	0.303	−0.131867	0.3570966
Time (min)	0.0037139	0.0031768	1.17	0.287	−0.0040595	0.0114874
Duration (week)	−0.0245532	0.0129349	−1.9	0.106	−0.0562039	0.0070974
_cons	1.441027	1.818227	0.79	0.458	−3.008015	5.890069

According to the sample population's age, exercise intensity, frequency, time, and duration, this study divided the research subjects into different subgroups, as shown in Table 4. The results from the subgroup analysis are as follows: (1) age: physical activity was beneficial for AD patients who were older than 75 years and had executive function issues. (2) Intensity: for executive function issues in AD patients, maintaining a 60–79 or an 80–89% maximal heart rate (MHR) during physical activity was more effective. (3) Frequency: the frequency of physical activity has a significant impact on the improvement of the executive function in AD patients, and the effect of 1–2 times/week was better than the effect of 3–4 or 5–7 times/week. (4) Time: a total of 60–150 min of physical activity per exercise session significantly improved executive function in AD patients. (5) Duration: an intervention duration of 16 or 24–48 weeks showed a significant effect on executive function issues in AD patients. (6) Event: for executive function issues in AD patients, both dual-task exercises and multimodal exercise had significant effects.

**Table 4:** Subgroup analysis of executive function issues in AD patients.

**Group**	**Subgroup**	***N***	**SMD**	**95% CI**	***p***	***I*^**2**^**
Age	65–75	5	0.44	0.109, 0.771	0.065	54.80%
	Older than 75	7	0.424	0.136, 0.713	0.086	45.80%
Intensity (%)	35–59	2	0.574	−0.356, 1.505	0.029	79.00%
	60–79	8	0.549	0.303, 0.794	0.293	17.40%
	80–89	2	0.134	−0.076, 0.343	0.968	0.00%
Frequency (times/week)	1–2 times/week	4	0.293	0.052, 0.534	0.403	0.00%
	3–4 times/week	7	0.473	0.155, 0.790	0.021	59.70%
	5–7 times/week	1	0.79	0.145, 1.435	—	—
Time (min)	30≤min<60	3	0.604	−0.023, 1.231	0.022	73.90%
	60≤min<90	7	0.424	0.136, 0.713	0.086	45.80%
	90≤min≤150	2	0.322	−0.049, 0.694	0.279	14.80%
Duration (week)	8–12 weeks	3	0.606	−0.033, 1.244	0.107	55.20%
	16 weeks	5	0.269	0.082, 0.692	0.047	44.80%
	24–48 weeks	4	0.387	0.051, 0.724	0.124	47.90%
Event	Single exercises	1	1.115	0.342, 1.888	—	—
	Dual-task exercises	3	0.492	−0.003, 0.988	0.144	48.3%
	Multimodal exercise	8	0.345	0.123, 0.567	0.111	40.1%

*“—” heterogeneity test cannot be conducted due to the lack of literature*.

#### Effect of Physical Activity on Working Memory Issues in AD Patients

**Figure 4:**
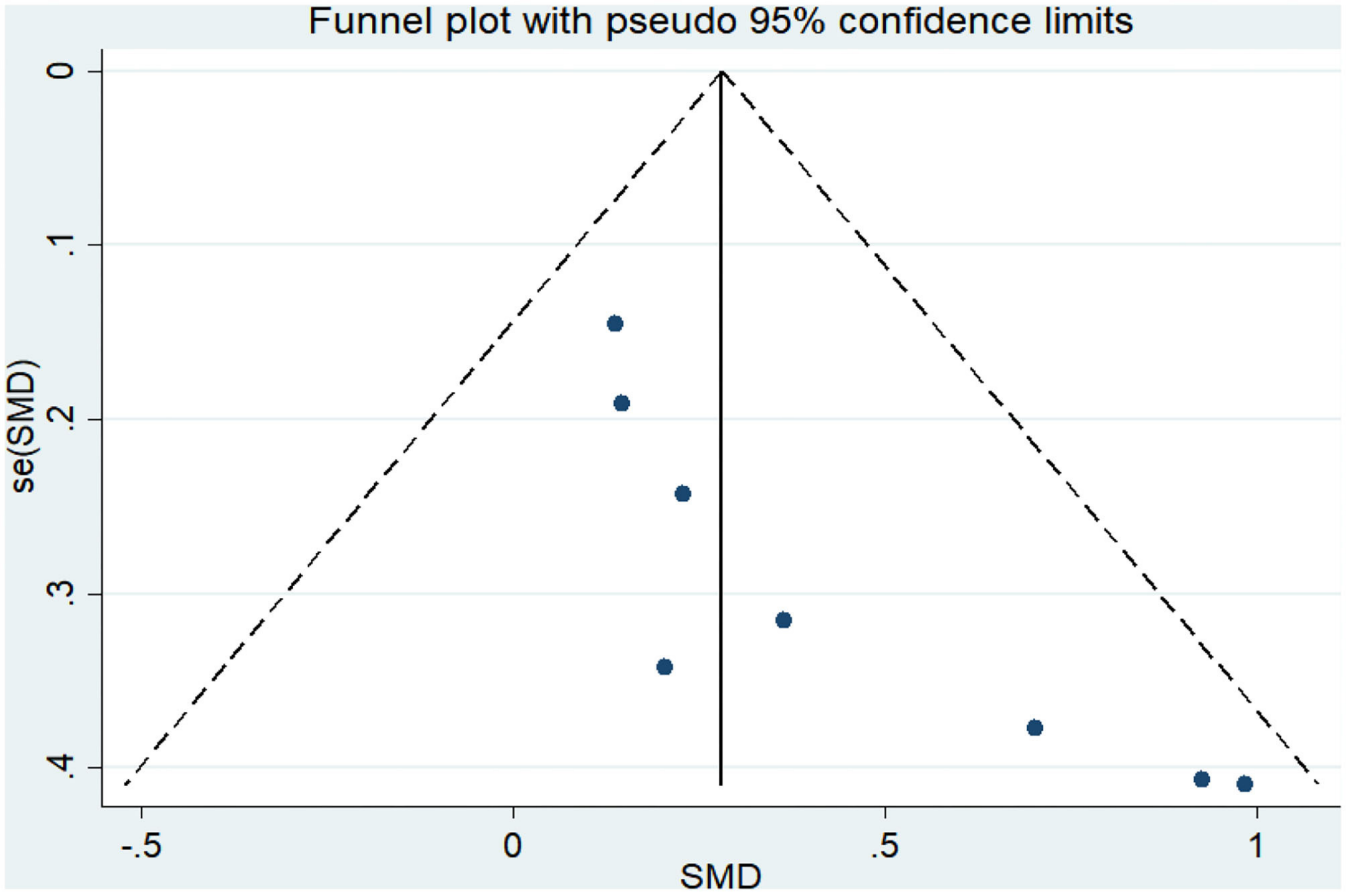
Funnel plot of publication bias for working memory.

**Figure 5:**
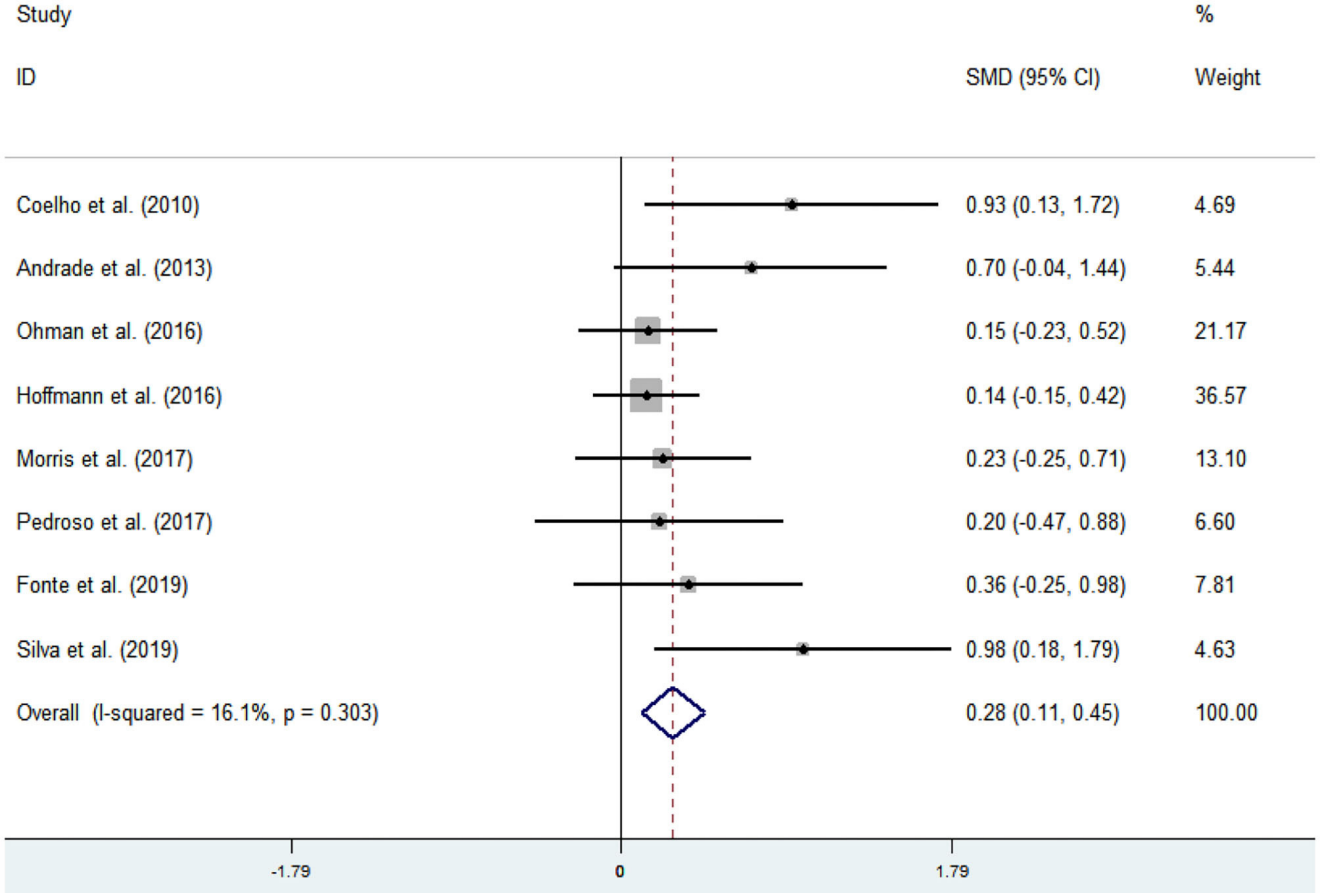
Effect of physical activity on working memory.

Eight articles (Coelho et al., 2012; Andrade et al., 2013; Hoffmanna et al., 2016; Ohman et al., 2016; Morris et al., 2017; Pedroso et al., 2017; Fonte et al., 2019; Silvaa et al., 2019) evaluated the effects of physical activity on working memory issues in AD patients. An asymmetrical funnel plot was presented. The funnel plot shows that there were no outlier values (Figure 4). The heterogeneity test results of the included research literature were not significant (*p* = 0.303, *I*^2^ = 16.1%); thus, the fixed-effects model was used for meta-analysis (Figure 5). The meta-analysis of eight studies demonstrated that physical activity had significant effects on improving working memory in AD patients (SMD = 0.28, 95% CI 0.11–0.45, *p* < 0.05).

The results of the regression of covariates for cognitive flexibility issues in AD patients are presented in Table 5. For cognitive flexibility, there were no significant effects of age (95% CI −0.1637734 to 0.2960517, *p* = 0.341), intensity (95% CI −0.2011265 to 0.2131423, *p* = 0.912), frequency (95% CI −1.843857 to 1.376603, *p* = 0.596), time (95% CI −0.0309679 to 0.0251433, *p* = 0.699), or duration (95% CI −0.1516738 to 0.1202981, *p* = 0.669).

**Table 5:** Covariate regression analysis of working memory issues in AD patients.

**_ES**	**Coef**.	**Std. err**.	***t***	***p* > *t***	**(95% CI)**	
Age	0.0661391	0.0534351	1.24	0.341	−0.1637734	0.2960517
Intensity (%)	0.0060079	0.0481411	0.12	0.912	−0.2011265	0.2131423
Frequency (times/week)	−0.2336269	0.3742413	−0.62	0.596	−1.843857	1.376603
Time (min)	−0.0029123	0.0065205	−0.45	0.699	−0.0309679	0.0251433
Duration (week)	−0.0156879	0.0316051	−0.5	0.669	−0.1516738	0.1202981
_cons	−3.939082	7.507129	−0.52	0.652	−36.23965	28.36149

All eligible studies were analyzed in subgroups based on age, intensity, frequency, time, and duration, as shown in Table 6. The results from subgroup analysis are as follows: (1) age: physical activity was beneficial for AD patients aged 65–75 years with working memory issues. (2) Intensity: for working memory issues in AD patients, maintaining a 60–79% MHR during physical activity was more effective. (3) Frequency: the frequency of physical activity has a significant impact on the improvement of the working memory issues in AD patients, and the effect of 3–4 times/week was better than the effect of 1–2 times/week. (4) Time: a total of 60–150 min per exercise session significantly improved working memory in AD patients. (5) Duration: an intervention duration of 16 or 24–48 weeks showed a significant effect on working memory issues in AD patients. (6) Event: for working memory issues in AD patients, dual-task exercises had a significant effect.

**Table 6:** Subgroup analysis of working memory issues in AD patients.

**Group**	**Subgroup**	***N***	**SMD**	**95% CI**	***p***	***I*^**2**^**
Age	65–75	2	0.161	−0.084, 0.406	0.748	0.00%
	Older than 75	6	0.397	0.154, 0.641	0.265	22.40%
Intensity (%)	35–59	1	0.146	−0.229, 0.521	—	—
	60–79	5	0.408	0.126, 0.689	0.534	0.00%
	80–89	2	0.233	−0.036, 0.502	0.051	73.70%
Frequency (times/week)	1–2 times/week	3	0.274	−0.003, 90.550	0.174	42.70%
	3–4 times/week	5	0.284	0.064, 0.505	0.303	17.50%
Time (min)	30≤min<60	1	0.137	−0.148, 0.423	−	−
	60≤min<90	5	0.404	0.139, 0.668	0.169	37.80%
	90≤min≤150	2	0.279	−0.098, 0.657	0.734	0.00%
Duration (week)	8–12 weeks	2	0.526	0.011, 1.041	0.144	53.10%
	16 weeks	3	0.282	0.030, 0.535	0.303	16.10%
	24–48 weeks	3	0.212	−0.054, 0.478	0.837	0.00%
Event	Dual-task exercises	3	0.491	−0.014, 0.995	0.165	46.7%
	Multimodal exercise	5	0.396	0.012, 0.780	0.038	60.7%

#### Effect of Physical Activity on Cognitive Flexibility Issues in AD Patients

**Figure 6:**
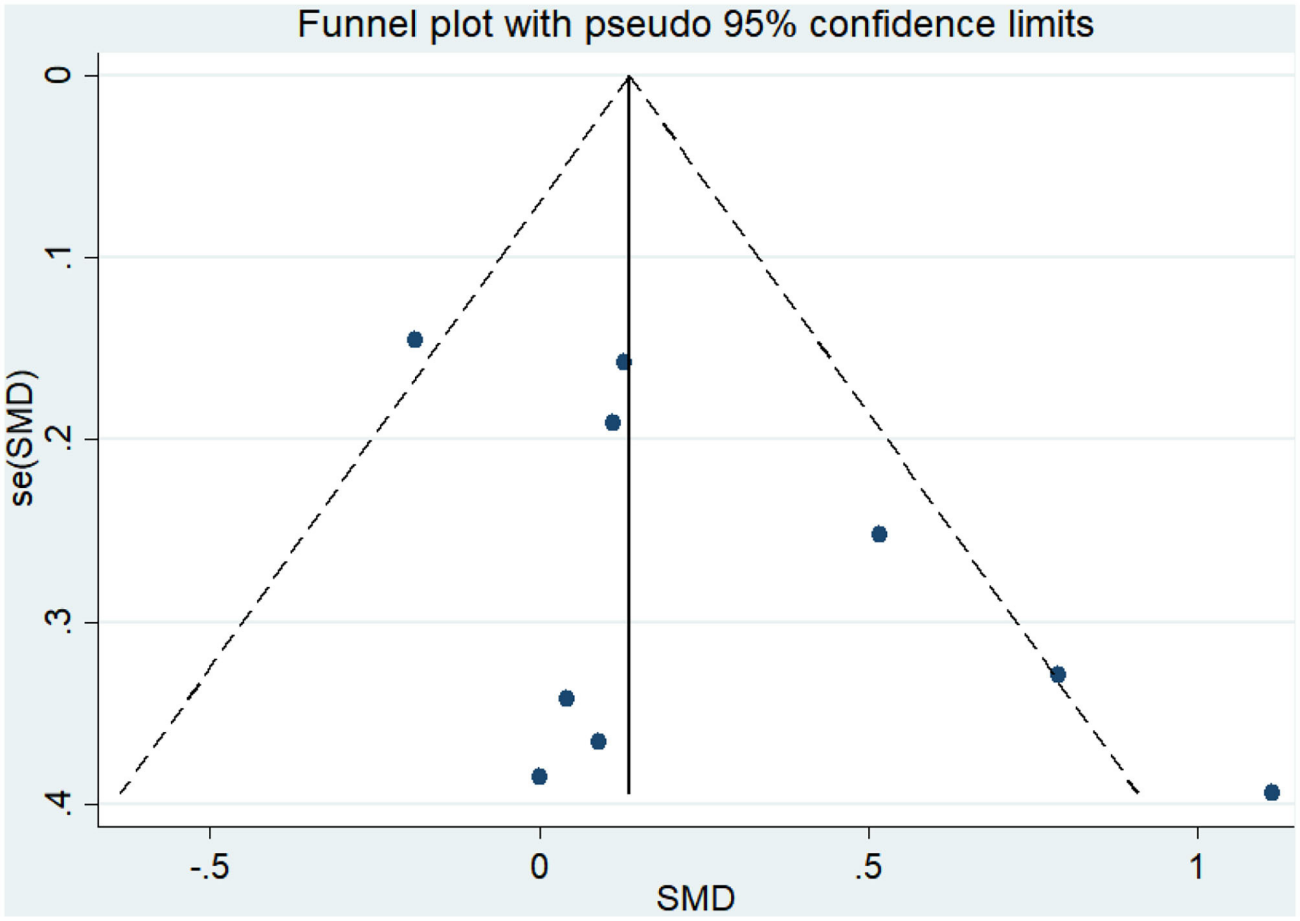
Funnel plot of publication bias for cognitive flexibility.

**Figure 7:**
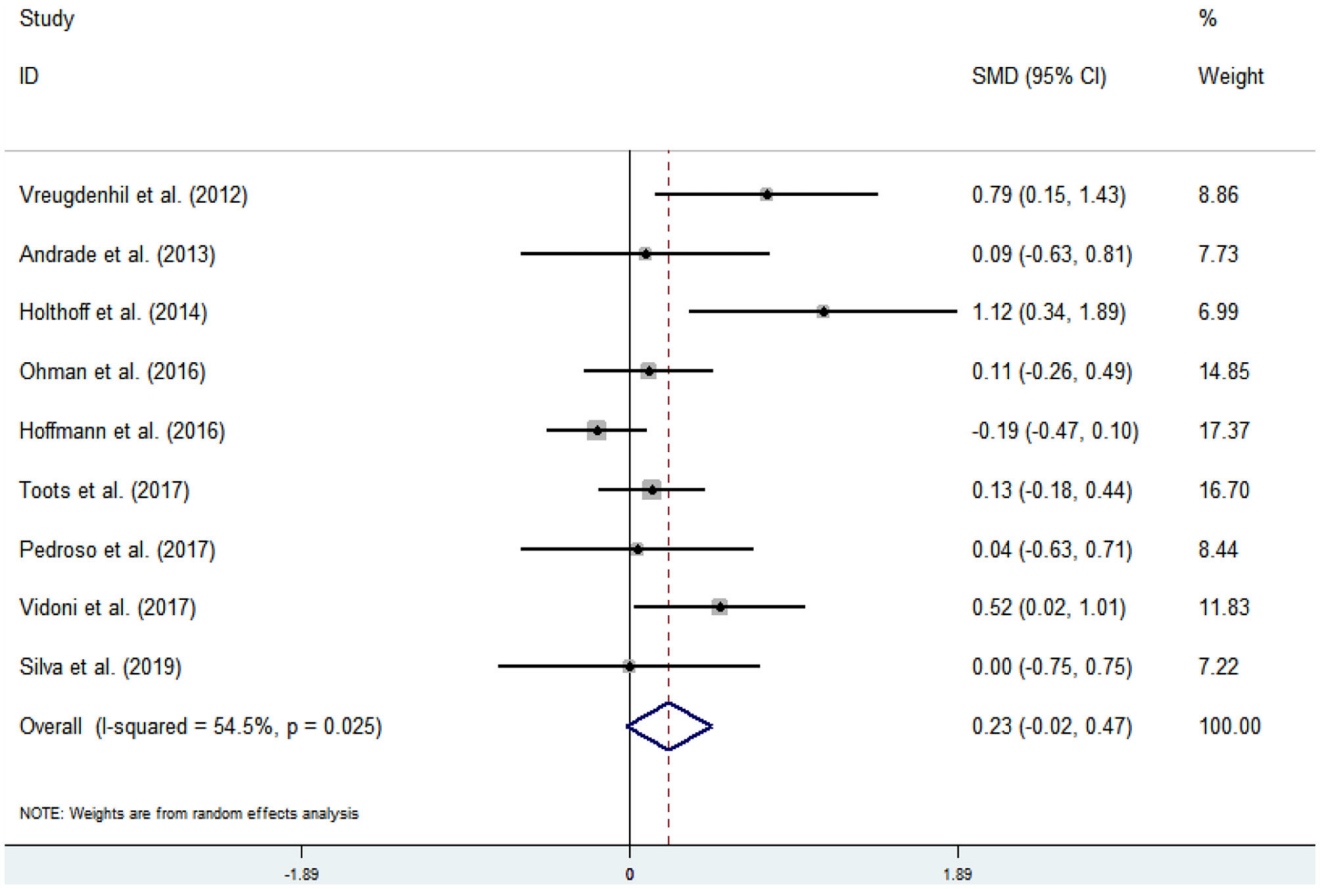
Effect of physical activity on cognitive flexibility.

Nine articles (Vreugdenhill et al., 2012; Andrade et al., 2013; Holthoff et al., 2015; Hoffmanna et al., 2016; Ohman et al., 2016; Pedroso et al., 2017; Tootsa et al., 2017; Vidoni et al., 2017; Silvaa et al., 2019) evaluated the effects of physical activity on cognitive flexibility issues in AD patients. An asymmetrical funnel plot was presented. According to the funnel plot, there were two outliers (Figure 6). There was a moderate heterogeneity in the research literature (*p* = 0.025, *I*^2^ = 54.5%), and the meta-analysis was performed using a random-effects models (Figure 7). Figure 7 shows that the meta-analysis of nine studies demonstrated that physical activity had a significant effect on improving cognitive flexibility in AD patients (SMD = 0.23, 95% CI −0.02 to 0.47, *p* < 0.01).

The analysis of heterogeneity and sensitivity showed that there was considerable bias in the studies by Vreugdenhill et al. (2012) and Holthoff et al. (2015). Therefore, a meta-analysis of the remaining RCTs was performed after exclusion. The results showed that the heterogeneity was reduced (*I*^2^ = 7.4%, *p* = 0.372, SMD = 0.06, 95% CI −0.11, 0.23), and that the differences between groups were significant (*p* < 0.01).

The results of the regression of covariates for working memory issues in AD patients are presented in Table 7. For working memory, there were no significant effects of age (95% CI −0.2086577 to 0.2332289, *p* = 0.608), intensity (95% CI −0.2536149 to 0.2638656, *p* = 0.843), frequency (95% CI −5.644876 to 5.888054, *p* = 0.833), time (95% CI −0.0894664 to 0.1045166, *p* = 0.505), or duration (95% CI −0.2578807 to 0.2722502, *p* = 0.789).

**Table 7:** Covariate regression analysis of cognitive flexibility issues in AD patients.

**_ES**	**Coef**.	**Std. err**.	***t***	***p* > *t***	**(95% CI)**	
Age	0.0122856	0.0173886	0.71	0.608	−0.2086577	0.2332289
Intensity (%)	0.0051253	0.0203633	0.25	0.843	−0.2536149	0.2638656
Frequency (times/week)	0.121589	0.4538306	0.27	0.833	−5.644876	5.888054
Time (min)	0.0075251	0.0076334	0.99	0.505	−0.0894664	0.1045166
Duration (week)	0.0071848	0.0208611	0.34	0.789	−0.2578807	0.2722502
_cons	−2.169054	2.459361	0.88	0.540	−33.4182	29.08009

The results from the subgroup analysis are as follows (Table 8): (1) age: physical activity was beneficial for cognitive flexibility issues in AD patients who were older than 75 years. (2) Intensity: for cognitive flexibility issues in AD patients, the effect of maintaining a 60–79 or 80–89% MHR was more effective. (3) Frequency: for cognitive flexibility issues in AD patients, the effect of performing physical activity 1–2 or 3–4 times/week was more effective. (4) Time: a total of 60–90 min per exercise session significantly improved cognitive flexibility in AD patients. (5) Duration: an intervention duration of 16 or 24–48 weeks showed a significant effect on cognitive flexibility issues in AD patients. (6) Event: for cognitive flexibility issues in AD patients, both dual-task exercises and multimodal exercise had significant effects.

**Table 8:** Subgroup analysis of cognitive flexibility issues in AD patients.

**Group**	**Subgroup**	***N***	**SMD**	**95% CI**	***p***	***I*^**2**^**
Age	65–75	2	−0.011	−0.258, 0.237	0.015	83.00%
	Older than 75	5	0.103	−0.104, 0.309	0.998	0.00%
Intensity (%)	35–59	1	0.112	−0.263, 0.487	−	−
	60–79	3	0.29	−0.058, 0.638	0.441	0.00%
	80–89	3	−0.039	−0.241, 0.163	0.336	8.30%
Frequency (times/week)	1–2 times/week	3	0.225	−0.053, 0.503	0.36	2.20%
	3–4 times/week	4	−0.025	−0.218, 0.168	0.506	0.00%
Time (min)	30≤ min <60	2	−0.042	−0.252, 0.168	0.141	53.90%
	60≤ min <90	4	0.082	−0.195, 0.359	0.994	0.00%
	90≤ min ≤ 150	1	0.518	0.024, 1.013	−	−
Duration (week)	8–12 weeks	2	0.424	−0.477, 0.525	0.174	56.80%
	16 weeks	3	−0.031	−0.233, 0.170	0.318	12.70%
	24–48 weeks	2	0.260	−0.039, 0.559	0.199	39.40%
Event	Dual-task exercises	2	0.107	−0.225, 0.440	0.960	0.00%
	Multimodal exercise	5	0.073	−0.175, 0.322	0.174	37.1%

#### Effect of Physical Activity on ADL Issues in AD Patients

**Figure 8:**
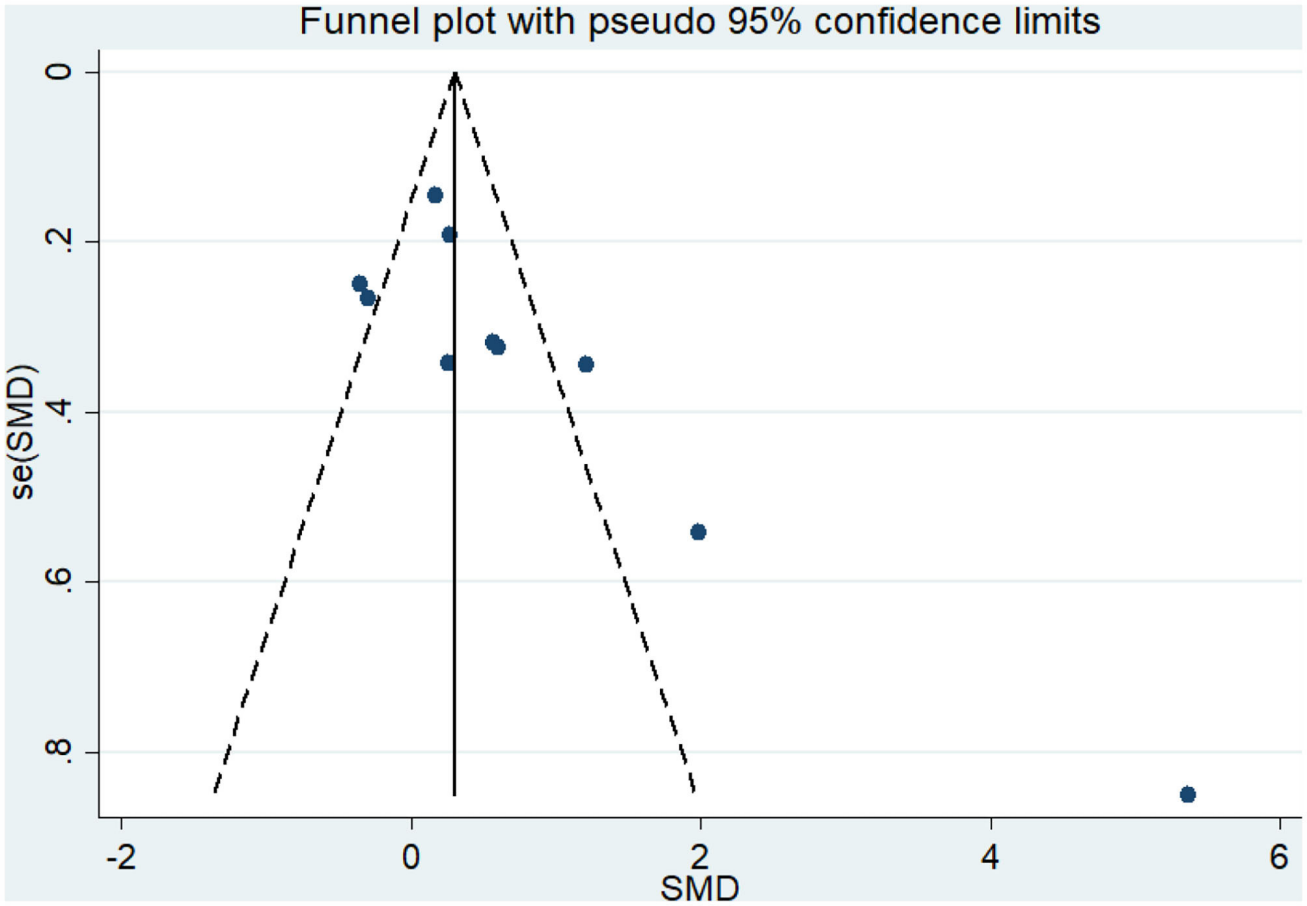
Funnel plot of publication bias for ADLs.

**Figure 9:**
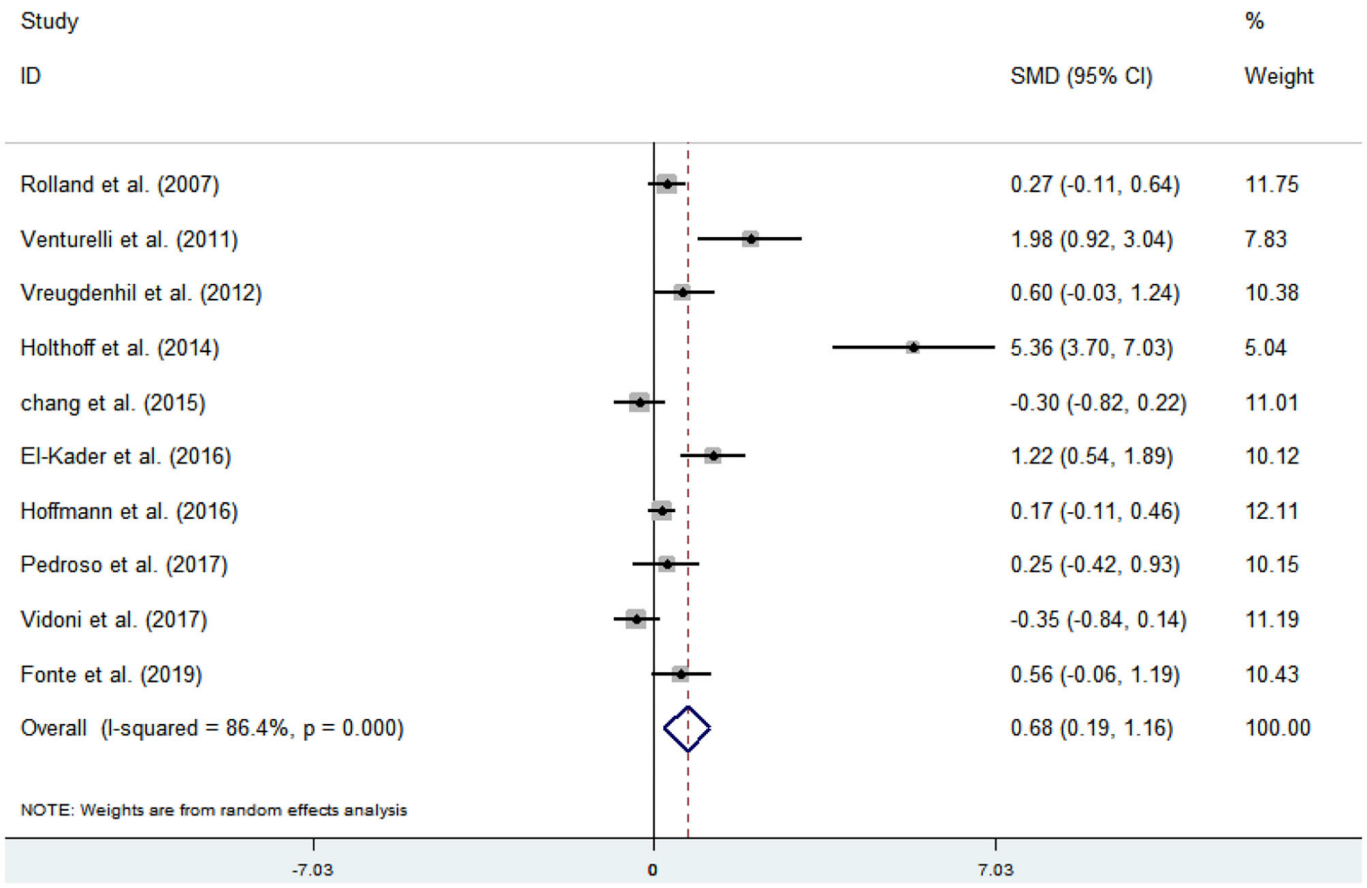
Effect of physical activity on ADLs.

Ten articles (Venturelli et al., 2011; Vreugdenhill et al., 2012; Chang et al., 2015; Holthoff et al., 2015; El-Kader and Al-Jiffr, 2016; Hoffmanna et al., 2016; Pedroso et al., 2017; Rolland et al., 2017; Vidoni et al., 2017; Fonte et al., 2019) evaluated the effects of physical activity on ADL issues in AD patients. An asymmetrical funnel plot was presented. Funnel plot indicated that there were three outliers (Figure 8). Heterogeneity testing of the study literature showed high heterogeneity (*p* < 0.000, *I*^2^ = 86.4%), and a random-effects model was used for the meta-analysis (Figure 9). Figure 9 shows that the meta-analysis of 10 studies demonstrated that physical activity had significant effects on improving ADLs in AD patients (SMD = 0.68, 95% CI 0.19–1.16, *p* < 0.001).

The analysis of heterogeneity and sensitivity showed that there was considerable bias in the studies by Venturelli et al. (2011), Holthoff et al. (2015), and El-Kader and Al-Jiffr (2016) thus, a meta-analysis of the remaining RCTs was performed after exclusion. The results showed that the heterogeneity was reduced (*I*^2^ = 45.1%, *p* = 0.091, SMD = 0.15, 95% CI −0.10, 0.39), and that the differences between groups were significant (*p* < 0.001).

The results of the regression of covariates for ADL issues in AD patients are presented in Table 9. For ADLs, there were no significant effects of age (95% CI −0.2683096 to 0.4846618, *p* = 0.47), intensity (95% CI −0.2702262 to 0.0794482, *p* = 0.204), frequency (95% CI −1.392111 to 1.053637, *p* = 0.72), time (95% CI −0.0623979 to 0.035701, *p* = 0.492), or duration (95% CI −0.2969797 to 0.113052, *p* = 0.281).

**Table 9:** Covariate regression analysis of ADL issues in AD patients.

**_ES**	**Coef**.	**Std. err**.	***t***	***p* > *t***	**(95% CI)**	
Age	0.1081761	0.1355999	0.8	0.47	−0.2683096	0.4846618
Intensity (%)	−0.095389	0.0629716	−1.51	0.204	−0.2702262	0.0794482
Frequency (times/week)	−0.1692369	0.4404458	−0.38	0.72	−1.392111	1.053637
Time (min)	−0.0133484	0.0176663	−0.76	0.492	−0.0623979	0.035701
Duration (week)	−0.0919639	0.0738411	−1.25	0.281	−0.2969797	0.113052
_cons	2.195003	9.602787	0.23	0.83	−24.46661	28.85662

The results from the subgroup analysis are as follows (Table 10): (1) age: physical activity was beneficial for ADL issues in AD patients who were older than 75 years. (2) Intensity: for ADL issues in AD patients, the effect of maintaining a 60–79% MHR was more effective. (3) Frequency: for ADL issues in AD patients, the effect of performing physical activity 3–4 times/week was more effective. (4) Time: a total of 30–60 or 60–90 min per exercise session significantly improved the ADL issues in AD patients. (5) Duration: for ADL issues in AD patients, the intervention duration was not important. (6) Event: for ADL issues in AD patients, multimodal exercise was significant.

**Table 10:** Subgroup analysis of ADL issues in AD patients.

**Group**	**Subgroup**	***N***	**SMD**	**95% CI**	***p***	***I*^**2**^**
Age	65–75	4	0.019	−0.358, 0.395	0.049	61.90%
	Older than 75	3	0.328	0.038, 0.618	0.708	0.00%
Intensity (%)	35–59	2	−0.02	−0.623, 0.582	0.05	73.90%
	60–79	4	0.255	−0.189, 0.699	0.096	52.70%
	80–89	1	0.172	−0.114, 0.458	−	−
Frequency (times/week)	1–2 times/week	2	−0.02	−0.623, 0.582	0.05	73.90%
	3–4 times/week	4	0.147	−0.158, 0.451	0.203	34.90%
	5–7 times/week	1	0.603	−0.031, 1.238	−	−
Time (min)	30≤ min <60	4	0.291	−0.087, 0.668	0.225	32.20%
	60≤ min <90	2	0.18	−0.159, 0.519	0.179	38.80%
	90 ≤ min ≤ 150	1	−0.349	−0.839, 0.141	−	−
Duration (week)	8–12 weeks	1	0.253	−0.42, 0.926	−	−
	16 weeks	3	0.137	−0.281, 0.554	0.091	58.30%
	24–48 weeks	3	0.142	−0.347, 0.632	0.049	66.80%
Event	Single exercises	1	−0.298	−0.821, 0.224	−	−
	Multimodal exercise	6	0.210	−0.033, 0.453	0.165	36.3%

*“−” heterogeneity test cannot be conducted due to the lack of literature*.

## Discussion

The purpose of this systematic review and meta-analysis was to compile and analyze the literature pertaining to RCTs on physical activity (e.g., aerobic exercise, aerobic fitness, walking, cycling, strength training, balance training, and flexibility training) in relation to its influence on executive function, working memory, cognitive flexibility, and ADLs among AD patients to determine an optimal exercise prescription. The results suggest that physical activity can improve executive function, working memory, cognitive flexibility, and ADL issues in AD patients.

This systematic review investigated the influence of physical activity on executive function and ADL issues in AD patients. Research suggests that the effect size of executive function was 0.22–0.62 (*p* < 0.05), that of working memory was 0.11–0.45 (*p* < 0.05), that of cognitive flexibility was −0.02 to 0.47 (*p* < 0.01), and that of ADLs was 0.19–1.16 (*p* < 0.001). Furthermore, significant heterogeneity may have been present in the study by Holthoff et al. (2015). In this study, physical activity was conducted on comfortable chairs to encourage patients to participate and prevent falls and other injuries. This method was different from those used in other studies and may be the cause of the heterogeneity.

The subgroup analysis showed that, for executive function issues, performing moderate-to-high-intensity dual-task exercises or multimodal exercise for more than 60 min per session for 16 weeks had a greater effect on AD patients. Regarding frequency, performing physical activity 1–2 times/week was found to be significant. In addition, for working memory and cognitive flexibility issues, performing 60–90 min of moderate-intensity dual-task exercises 1–4 times/week was more effective. The subgroup analysis indicated that, for ADL issues, maintaining a 60–79% MHR during multimodal exercise 3–4 times/week for 30–90 min each time had a greater effect on AD patients. In addition, in the subgroup analyses of executive function, working memory, cognitive flexibility, and ADL issues, maintaining a 35–59% MHR during physical activity did not show a strong effect compared with maintaining a 60–79 or an 80–89% MHR. Performing physical activity 1–4 times/week for 60–90 min per exercise session significantly improved executive function, working memory, cognitive flexibility, and ADL issues in AD patients. In addition, this meta-analysis provides evidence to support the inclusion of aerobic training and strength, flexibility, balance, and other physical activities to improve executive function and ADL issues in AD patients. For AD patients with executive function, working memory, cognitive flexibility, and ADL issues, both dual-task exercises and multimodal exercises had significant effects. Notably, for working memory and cognitive flexibility issues, dual-task exercises showed a strong influence compared with multimodal exercises. Coincidentally, our findings support the WHO recommendations (World Health Organization Physical Activity Older Adults, 2017). In addition, the characteristics of exercise intervention in the included studies support the frequency, intensity, and time per session recommended by the WHO, but slightly lower than the WHO recommendations based on expert advice. Our meta-analysis showed that the current WHO recommendations can be considered an effective exercise prescription for AD patients; however, future studies are needed to determine which combinations of intervention methods, intensity, duration of each intervention session, frequency, and duration best improve executive function and ADL issues among older adults diagnosed with AD.

Systematic reviews showed that physical activity can improve the attention, cognitive function, executive function, and language of AD patients (Coelho et al., 2009; Farina et al., 2014). These authors agree that there is not enough theoretical support for the ideal intervention program, and that there is no consensus on the intensity, frequency, and duration of exercise. However, long-term physical activity stimulates growth factors, neurotransmitter synthesis, oxygenation, and plasticity to produce nerve and neuroprotective effects on the brain (Deslandes et al., 2009). The beneficial effects of physical activity on brain aging or dementia have not been well-documented (Herholz et al., 2013). However, animal studies have revealed that the activation of adult neurogenesis (Kempermann et al., 2010) or the increase in plasma levels of neuroplasticity-related brain-derived neurotrophic factors (Coelho et al., 2014) is related to physical activity. Furthermore, some mechanisms related to physical activity help to improve cognitive function, such as improvement of the nervous system, including promoting the synthesis of neurotransmitters and improving cerebral blood flow (Eggermont et al., 2006; Lista and Sorrentin, 2009). Some studies have shown that physical activity can increase brain-derived neurotrophic factor and also have a positive impact on the brain neuroplasticity. Kramer and Erickson found that in rodents, physical activity induces increased levels of brain-derived neurotrophic factor in the frontal cortex, hippocampus, and cerebellum and promotes the formation of new capillaries in these areas (Kramer and Erickson, 2007). Some authors believe that physical activity improves cerebral circulation by increasing cerebral blood flow and oxygen supply (Hamer and Chid, 2009; Sofifi et al., 2011), while promoting cardiovascular and cerebrovascular health by lowering blood pressure and blood lipid levels. Physical activity also promotes inflammatory markers and can effectively enhance endothelial function (Kivipelto et al., 2005). Furthermore, physical activity can stimulate the proliferation of neurons in the hippocampus (Erickson et al., 2011). Therefore, the effect of physical activity on clinical performance in AD (e.g., cognitive and executive functions) may rely on improvement in brain functionality.

Previous studies have shown that physical activity can improve the cognitive and executive functions of the elderly with cognitive impairment (Scherder et al., 2005; Lautenschlager et al., 2008; Uffelen et al., 2008; Baker et al., 2010; Lam et al., 2011). The executive function is mainly responsible for the self-regulation of behavior and is the key cognitive resource, including the ability to initiate, plan, sequence, and monitor (Miyake and Friedma, 2012). In AD patients, executive dysfunction is a prominent clinical symptom that directly affects the patient's self-regulation of behavior and ADLs (Boyle et al., 2003; Razani et al., 2007).

The promoting effect of non-pharmacological treatment methods on the improvement of the condition of AD patients has been confirmed (Morley and Silve, 1995; Cohen-Mansfifield and Mintze, 2005). Interventions, such as physical activity, have been shown to improve cognitive function and executive function and are even effective for frail residents of nursing homes (Lazowski et al., 1999). At the same time, exercise intervention may produce effective protective mechanisms to prevent the decline in the ADLs. At present, it has been confirmed that suitable physical activities can inhibit the pathophysiological changes of AD by regulating the expression and hydrolysis of amyloid precursor protein, reducing the production of β amyloid protein, and scavenging free radicals, among other actions (Radak et al., 2010; Foster et al., 2011). In addition, on the one hand, the decline of ADLs among AD patients is due to a decline in the cognitive level; on the other hand, it is closely related to the atrophy of muscles with the development of age and disease (Sakuma and Yamaguch, 2011). Physical activity is an important measure to combat skeletal muscle atrophy. It can increase the quality of skeletal muscle, output power of physical activity per unit time, and skeletal muscle endurance. According to the changes in muscle stimulation during physical activity, the number of activated transverse bridges can be increased, thereby enhancing muscle flexibility (Jin et al., 2020). Furthermore, the contraction ability of respiratory muscles is enhanced after exercise, which is conducive to the extension of patient's trunk and limbs, and provides opportunities for improving limb coordination and strengthening exercise ability (Liu et al., 2018). Therefore, physical activity can improve ADLs in AD patients by increasing the quality of skeletal muscle, enhancing muscle activity, improving limb coordination, and exercise performance, etc.

In this review, one article showed that there was no significant effect of physical activity on executive function among AD patients. Specifically, Morris et al. (2017) showed that there was no significant difference in the improvement of executive function between the intervention group and the control group. Aerobic exercise interventions were used in this study, which are different from other interventions. This may be one of the reasons for the lack of a significant difference. However, aerobic exercise interventions were also used in the study by Vidoni et al. (2017), and the results showed a significantly different effect on ADL issues in AD patients. This result suggests that aerobic exercise may be more effective in improving the quality and activity of skeletal muscle in AD patients. In general, more RCTs are required to demonstrate the effect of aerobic exercise on executive function and ADL issues in AD patients. In addition, other studies have shown that physical activity can improve executive function and ADL issues in AD patients. Therefore, physical activity is feasible and may provide an alternative or adjuvant treatment for patients with mild to moderate or even late AD and their family nurses.

## Limitations

There are some limitations and deficiencies in this study. The authenticity and reliability of the results are affected by the course of disease and the frequency, time, intensity, and duration of the intervention in AD patients. There are certain differences in the quality of the eligible articles for our study: (1) only four studies showed their concealed allocation, which may be one of the reasons for the systematic bias of results (PEDro-scale, 2010). (2) Only four articles referred to blinding of assessors. (3) Most of the studies lacked a description of the course of the disease, which could have led to a lack of understanding of the effectiveness of physical activity on executive function and ADL issues in AD patients. (4) Most studies did not mention the severity of disease among patients with AD, which made it impossible to provide patients with more targeted exercise prescriptions. (5) Only two authors, Hoffmanna et al. (2016) and Silvaa et al. (2019), mentioned the effect of physical activity on inhibitory functions in AD patients. Studies on inhibitory executive functions among AD patients are scarce and were not included in the meta-analysis for the time being to avoid errors. Furthermore, the differences in experimental intensity, time, frequency, duration, and outcome measure methods could have led to differences in outcomes and caused difficulty in explanations.

## Conclusions

Research has proven that physical activity can effectively improve executive function, working memory, cognitive flexibility, and ADL issues in AD patients and may be an alternative or auxiliary treatment. In the future, it will necessary to provide more accurate neuropsychological evaluations of each executive function, working memory, cognitive flexibility, and ADL dimension and to grade the course of AD to obtain more accurate exercise prescriptions for physical activity interventions. In the absence of adverse reactions, medical profession could combine physical activity with daily medical treatment to optimize the treatment of executive function, working memory, cognitive flexibility, and performance of ADLs in AD patients. In addition, the conclusion of this study still needs to be confirmed by a high-quality large-sample RCT.

## Data Availability Statement

The original contributions presented in the study are included in the article/supplementary materials, further inquiries can be directed to the corresponding author/s.

## Author Contributions

LW and LZ contributed to the idea and structural design plan for review. XJ and HZ applied the search strategy. LZ, LW, and LL applied the selection criteria to screen out qualified documents. LW and XJ completed the deviation risk assessment. LZ wrote the manuscript. LW, LL, and HZ edited the manuscript. All authors analyzed and interpreted the data. All authors have read the complete manuscript and reached a consensus on the manuscript version.

## Conflict of Interest

The authors declare that the research was conducted in the absence of any commercial or financial relationships that could be construed as a potential conflict of interest.
